# OLDER ADULT WOMEN’S MULTIDIMENSIONAL RESPONSIBILITIES IN SKIPPED GENERATION HOUSEHOLDS IN NIGERIA

**DOI:** 10.1093/geroni/igz038.145

**Published:** 2019-11-08

**Authors:** Mojirayo Afolabi

**Affiliations:** Obafemi Awolowo University, Ile-Ife, Osun State, Nigeria

## Abstract

This contribution studies the roles of grandparents in caring for grandchildren in skipped generation households, from gender perspective. Historical studies often focus on health and economic status of older adults generally, without distinguishing older adult women whose responsibilities are often undervalued. Such assessments assume that both grandparents engage in caring for grandchildren, being the joy of old age. Highlighting women’s roles will ensure proper design and implementation of policies to enhance improvement in overall well-being of skipped generation households in Nigeria. The economic value of this is high. Using detailed data from three major states in Nigeria, - Imo, Lagos and Kano (representing each of the major ethnic groups) this study provides a detailed picture of the areas of women’s responsibilities in skipped generation households, using both quantitative and qualitative methodologies. The quantitative study employed structured questionnaire to collect primary data while the qualitative technique employed the use of in-depth interviews and focus group discussions. The older adult participants are grouped into three; less than sixty five years, between sixty five and eighty and those above eighty years of age. The study reveals that social norms and expectations impact the lives of older adults, ensuring that responsibilities sharing in skipped generation households are strongly impacted by gender roles. The paper concludes that in order to ameliorate the difficulties and challenges faced in performing these roles, governments and other organizations need to put the realities in the skipped generation households into consideration during planning processes.

